# The Nude Mouse as Model for Liver Deficiency Study and Treatment and Xenotransplantation

**DOI:** 10.1155/2012/140147

**Published:** 2012-10-31

**Authors:** Isabelle Vidal, Lysiane Richert

**Affiliations:** ^1^Faculté de Médecine et de Pharmacie, EA 3921, IFR 133, 25030 Besançon, France; ^2^Unit of Pediatric Surgery, Geneva University Hospital, 1211 Geneva, Switzerland; ^3^KaLy-Cell, 20A rue du Général Leclerc, 67115 Plobsheim, France

## Abstract

We aimed at reviewing the various uses of Nude mouse for the development of liver deficiency models and evaluation of efficacy of hepatic cell xenotransplantation. The first part records the large range of liver deficiency models that can be developed in Nude mice: surgical partial hepatectomy, acute toxic liver deficiency, chronic cirrhosis, and transgenic liver injury. The second part tackles the outcome of rat hepatocyte as well as human cell transplantation, both mature hepatocyte and hepatic progenitor, into Nude mouse submitted to liver injury. Results are discussed and compared to other available immunodeficient mouse models. The issue of humanized liver creation is also addressed. Altogether, these results show that Nude mouse appears to be a suitable small animal model to expand our insight into liver cell engraftment and regeneration.

## 1. Introduction

Orthotopic liver transplant remains the treatment of choice for fulminant or acute liver failure and end-stage chronic liver deficiency. Etiologies of these liver failures are numerous, ranging from metabolic liver diseases, infectious causes, nonlife compatible large hepatectomy to alcohol hepatic pathology and others. Most remain symptomatic treatments, and liver transplant is often the only key solution. Unfortunately, the increased need for organ transplantation is met by a lack of organ donors. Some surgical techniques such as living donor procedure [1] or cadaveric donor liver split sharing between two recipients [2]; as well as bioartificial hepatic support [3] can partly alleviate this shortage. 

The lack of therapeutic alternatives led scientists to develop cell transplantation research. Cell transplantation could not only replace solid organ transplantation for the medical indications listed above but also it could, in addition, be proposed for gene therapy applications, in which organ transplant is a procedure too heavy to be ethically accepted such as congenital deficiency of a liver metabolic pathway, that does not impair liver function.

We first focused on the evaluation of adult hepatocyte transplantation for treatment of liver deficiency by cell therapy [4, 5], since it presented many attractive aspects when compared to organ transplant: better availability of cells, less invasive surgery, redo possibility, and lesser cost. It could also become a bridge between fulminant liver deficiency and organ transplant, to gain time before getting an organ suitable for transplantation [6, 7].

More recently, liver stem cells appeared to also be good candidates for transplantation, with the advantage of being maintained longer in culture than mature hepatocytes which lose their functions after a few days and being easily harvested from various human sources (adult, embryonic and fetal liver, and nonhepatic mesenchymal tissue, as well as induced pluripotent stem cells (iPS)) [5, 8, 9]. 

To optimize understanding of cell transplantation mechanisms, small animal models were created to allow the study of cell engraftment conditions and mechanisms of liver regeneration. 

On the other hand, the major species differences in the metabolism of drugs require metabolism and toxicity studies using models as close as possible to humans. In addition to the current gold standard in vitro approach of cultured adult human hepatocytes for the prediction of in vivo hepatic metabolism, pharmacokinetic and toxicity in humans [10, 11], the use of in vivo approach of animals with humanized livers have been explored [12–14]. Furthermore, viral pathobiological studies, that is, hepatitis B/C viral infections and treatment that are not possible on primary human hepatocyte in culture since the cells become nonpermissive for HB/CV after plating, have been made possible with the development of animal models with humanized livers [15–17]. 

For these two research area applications, immunosuppressed animal models, that would bear xenogenic cell transplantation without immunosuppressive drug, needed to be developed. In order to allow the evaluation of treatment of liver deficiency, an additional request of animal models was that they could be submitted to treatments mimicking human clinical liver pathologies requiring cell transplant therapy. 

The vast majority of research began with murine models, such as immunodeficient mice, with normal liver, or undergoing liver failure (by toxic liver injury, partial hepatectomy, or transgenic modification) and was tested for xenogenic hepatocyte transplantation, at first murine-murine models secondarily extended to human-murine transplantation (Table 1).

A variety of immunosuppressed mouse models have been developed [32]. For the evaluation of liver deficiency treatment via cell transplantation, the most commonly used models are the SCID mouse, lacking functional T and B cells, and RAG2 mouse (transgenic mouse with recombinant activation gene-2 (RAG-2)s), which lack mature T and B lymphocytes [33]. Literature reports less numerous studies with Nude mice. Yet, this athymic mouse with high deficit of mature T cells due to a Fox gene family mutation (Foxn1 [34, 35]) is a more robust model than SCID or RAG2 mouse, and it can be bred in less immunoprotective environment [36, 37].

Nude mice have been used since 1968 [32] to study xenogenic tumor development [38–42], imaging testing [43], or gonadal grafts [44].

## 2. Nude Mouse Is Successful for Induction of Liver Injury 

In murine models, different liver injuries can be performed, to induce hepatic deficiency comparable to human's one: (1) surgical, by extensive partial hepatectomy; (2) toxic by administration of carbon tetrachloride (CCl_4_) (that can also act on transplanted hepatocyte because of its long half- life) [45–47], by D-Galactosamine that generates at low dose an apoptosis and at higher dose a necrosis and fulminant hepatitis [48], by retrorsine [49–51] that suppresses hepatocyte proliferation, or by Fas-ligand agonist that will trigger cell apoptosis on mouse hepatocyte, such as Jo2 antibody treatment [52–57]; (3) radiotherapy was also used to block liver regeneration [6, 58]. But, the most growth selective advantage model used is (4) transgenic mice, with natural liver injury by hepatotoxicity of metabolism disorders. There are two main models: uPA^+/+^ (uroplasminogen activator) mouse [59, 60] and Fah^−/−^ [61] (fumarylacetoacetate hydrolase) mouse. Interestingly, we have found that most of these liver injuries can be applied to Nude mice.

### 2.1. Surgical Partial Hepatectomy in Nude Mouse

In our experience [62], and in contrast to the more resistant wild-type Balb/c mice, Nude mice did not survive 70% partial hepatectomy performed according to the technique of Higgins and Anderson [63] and died within 24 h following the surgery. Our finding is not in accordance with the report of Strom et al. [64] referring to a high survival rate of Nude mice submitted to two third partial hepatectomy. Forty percent partial hepatectomy was successful and consisted, after median laparotomy, in the resection of the left lateral liver lobe of Nude mice, after ligation using 3 to 5 titanium 3-mm clips for stapling (LIGACLIPrm, Ethicon, France). Hemostasis was performed by dabbing the bloody cut surface with caution using a hemostatic cellulose gauze (SURGICELrm, Johnson & Johnson, France), with removal of this gauze, to decrease postoperative mortality [62]. We recommend the surgery not to exceed 5 min. After partial hepatectomy, animals were warmed up for at least 20 min under heating lamp to restore their preoperative body temperature and were supplemented for 24 h with 20% glucose in drinking water. 

Serum ALT increased (×2 compared with the day 0 value) on day 1 following surgery and returned to basal levels within 3 days. Histological features of liver regeneration, Ki67 and caspase 3 expression, proved that both mechanisms of apoptosis and cell proliferation coexist without any necrotic stage during liver regeneration [62]. This confirms literature data [65]. Restoration of liver mass occurred within 10 days.

### 2.2. Acute Toxic Liver Injury in Nude Mouse

#### 2.2.1. Thioacetamide (TA)

Various doses were tested on Nude mice, with a 20%-mortality at day 10 following an injection of 1700 mg/kg and 100%-mortality at 48 hours with a 2000 mg/kg TA dose. Serum ALT values reached 20 times the day 0 values 24 hours after a 1700 mg/kg TA injection to Nude mice; then a progressive return to basal values was observed, which was complete on day 7. This correlated with a massive liver necrosis (up to one-third of liver), maximal at 24 hours and then decreasing with normal histology at day 10 [62]. 

#### 2.2.2. Jo2 Single Dose

Fas antigen is a cell surface receptor that mediates cell apoptosis when stimulated. Jo2 antibody is a Fas-ligand agonist and acts by stimulating Fas antigen and cell apoptosis. Jo2 is mouse specific; thus, it does not trigger apoptosis of other species, and it acts mainly on hepatocyte [66]. Heart, lung, bone marrow, and kidney express Fas, but are not sensitive to Fas apoptosis [67].

**Figure 1:**
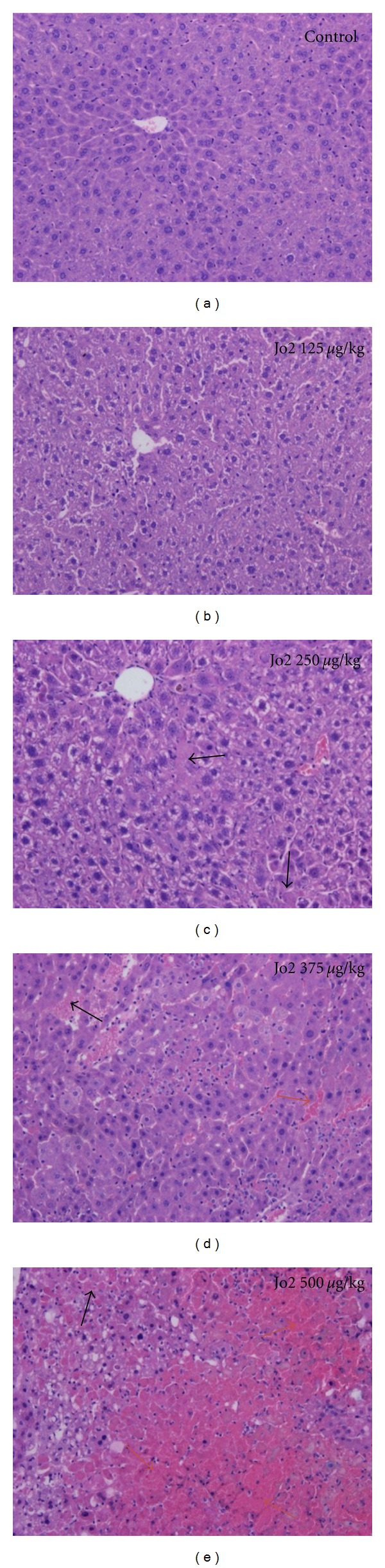
Nude mouse liver histology after hematoxylin and eosin staining, in mouse control (a) and after a single Jo2 injection of 125 *μ*g/kg (b), 250 *μ*g/kg (c), 375 *μ*g/kg (d), and 500 *μ*g/kg (e). Photomicrographs were taken using Olympus DP70 microscope with an original magnification of 100x. Black and blue arrows indicate apoptotic foci and necrotic foci, respectively.

The specific anti-mouse Fas monoclonal antibody Jo2 was injected at 125, 250, 375, or 500 *μ*g/kg IP dose to Nude mice, with higher sensitivity and mortality compared to Balb/c mice [62]. This anti-Fas antibody Jo2 was proven to induce fulminant hepatic failure in mice after a single injection [68–70], characterized by an elevation of serological ALT for the first three days. The survival rates correlated with histological observations of liver necrosis. Histologically, massive necrosis (with less than 5% of healthy parenchyma) was observed in Nude mice at 500 *μ*g/kg Jo2. Severe panlobular and panacinar necrosis already occurred at 375 *μ*g/kg Jo2 on day 1 after treatment (Figure 1). Liver deficiency injury was a dose-dependent effect, and it was reversible within 10 days. The regenerative capacity of the liver was thus retained after Jo2 injection, making its use attractive for studying the efficacy of hepatocyte transplantation [62].

#### 2.2.3. D-Galactosamine (GalN)

Nowak et al. [21] described a single intraperitoneal administration of 0.7 g/kg body weight D-Galactosamine, eventually followed 36 hours later by cell transplantation. The mortality rate was 62% in absence of transplantation, occurring in the first 72 hours.

#### 2.2.4. Carbon Tetrachloride (CCl_4_)

CCl_4_ was also used to induce acute liver deficiency in Nude mouse [23, 24] by intraperitoneal injection of 100 *μ*L/20 g body weight of 10% CCl_4_ in olive oil.

### 2.3. Induction of Liver Cirrhosis in Nude Mouse

**Figure 2:**
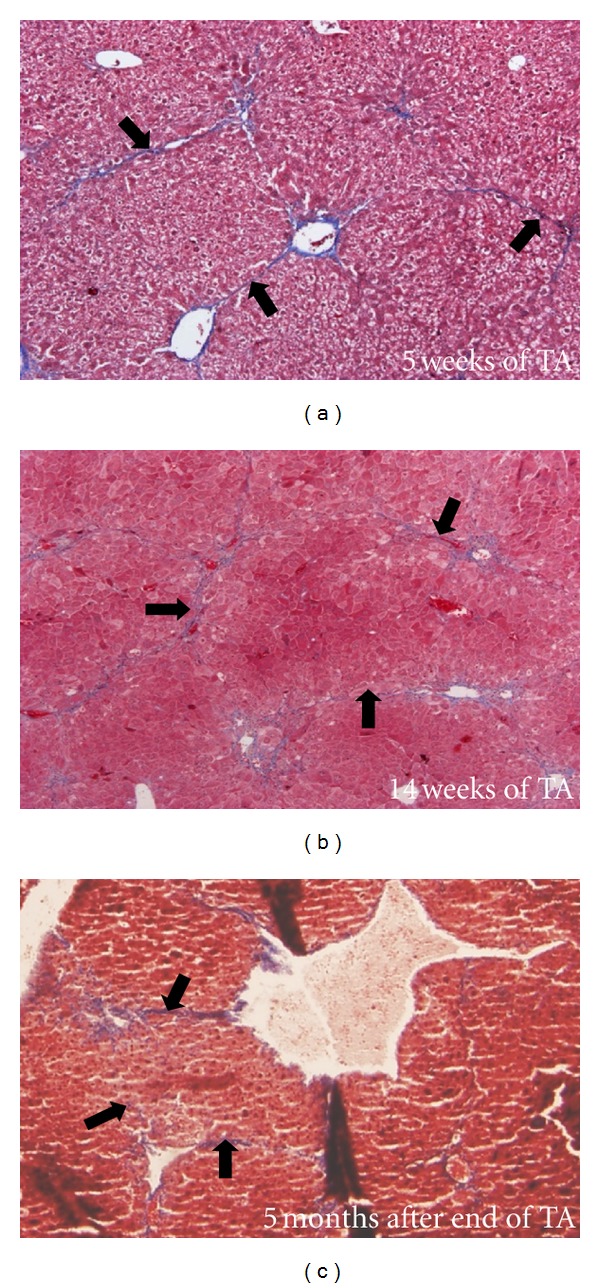
Liver histology after trichrome staining of Nude mice 5 weeks (a) and 14 weeks after the beginning of the thioacetamide cirrhogenic treatment and 5 months after the end of the 14-week-long treatment (c). Black arrows indicate fibrous extension. Photomicrographs were taken using Olympus DP70 microscope with an original magnification of 40x.

We succeeded in inducing cirrhosis in Nude mice, by repeated thioacetamide injections at 200 mg/kg of body weight, three times a week for 14 weeks [62]. Macroscopically, the liver of Nude mice presented a slightly rough surface after 5–8 weeks of treatment. Nude mice exhibited a spectacular fibrosis and onset of cirrhosis from 5 weeks of treatment. Histological features showed accentuated lobulation, with some nearly complete rings of connective tissue surrounding lobules (Figure 2). Micronodular cirrhosis was observed after 14 weeks. Ki67-positive cells were more numerous after 14 weeks of treatment compared with 5 weeks of treatment, reflecting intense proliferation of hepatocytes. Induction of cirrhosis in Nude mice was found to reverse to fibrosis within 5 months after cessation of the treatment [62]. 

### 2.4. uPA^+/+^ (Uroplasminogen Activator) Transgenic Nude Mouse

uPA-Nude mice were used by Rhim et al. [19] by crossing Alb-uPA transgenic mice with athymic nu/nu mice. This uPA^+/+^ mouse model presents an activation of the transgene that expresses uroplasminogen activator under the control of an albumin promoter. This overexpression of uPA causes liver injury with accumulation of hepatotoxic substrate and progressive depletion of hepatocytes, neonatal bleeding, associated to kidney disease. The homozygous mice die from liver deficiency, unless they received safe non-uPA hepatocyte transplantation. In heterozygous mice, population of non-uPA hepatocyte can spontaneously develop and repopulate the liver in about 8 weeks [59, 60]. It has been reported that hepatocytes without uPA expression, when transplanted, have a growth advantage over the hepatocytes with transgenic uPA^+/+^ leading to cell death [59, 60, 71, 72]. 

### 2.5. Fah^−/−^ (Fumarylacetoacetate Hydrolase) Transgenic Nude Mouse

The transgenic mouse model Fah^−/−^ is a mutant mouse deficient in tyrosine catabolic enzyme fumarylacetoacetate hydrolase, corresponding to clinical model of human type 1 tyrosinemia. This pathology results in progressive hepatocellular injury and mouse death in a few weeks. This liver injury can be prevented by a drug: 2-(2-nitro-4-trifluoromethylbenzoyl)-1,3-cyclohexanedione (NTBC), usually put in animal drinking water [61]. Breeding is easier than for uPA^+/+^ mice; there is no renal deficiency, and transplant can be performed at any time [27]. Azuma et al. used Fah^−/−^ Nude mouse model to test for human hepatocyte transplantation [27].

So, Nude mouse is a suitable small animal model in which it is possible to induce different types of hepatic deficiencies, from acute fulminant hepatitis to chronic cirrhosis, including surgical partial hepatectomy, and inborn inherited metabolic disorders. It also presents the advantage of an inborn immunosuppressed status, which could allow for xenogenic organ or cell transplantation. Thus, it was developed with different hepatic mature and progenitor cell transplantation, isolated from rat and human livers.

## 3. Nude Mouse Is a Successful Xenotransplantation Model for the Evaluation of the Efficacy of Cell Therapy

Before human clinical application of hepatocyte transplantation, it is of high importance to evaluate efficacy of cell transplantation on small animal models, in clinical situation. 

### 3.1. Nude Mouse Is a Successful Murine Xenotransplantation Model

As reported in literature [49, 73], engraftment of hepatocyte into recipient livers is largely favored if a selective advantage (i.e., existence of growth and proliferation difference) between donor and recipient hepatocytes does exist. This was confirmed in Nude mouse model transplanted with rat hepatocytes: on healthy liver, mature hepatocyte could engraft but with a very limited proliferative activity, and engraftment after 40% hepatectomy in Nude mice liver parenchyma did not differ from non-operated control Nude mice [62]. These results could be due to the time difference between transplanted rat hepatocyte and mouse hepatocyte proliferation, the peak of DNA synthesis being observed 24 hours after partial hepatectomy in the rat and 48 hours after partial hepatectomy in the mouse [74]. Neither a single dose of Jo2 pretransplantation treatment nor TA-liver injury improved rat hepatocyte engraftment [62]. 

**Figure 3:**
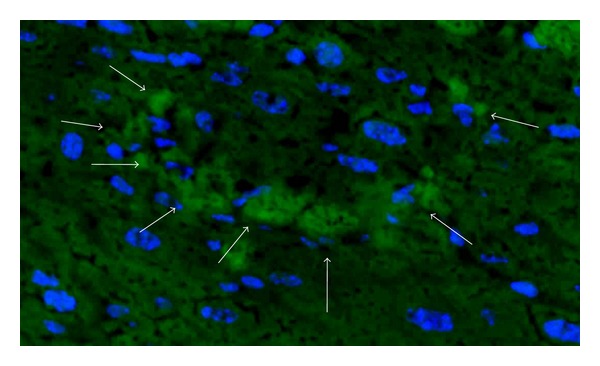
Immunodetection of engrafted rat hepatocytes in Nude mouse liver (original magnification ×40), 21 days after cell transplantation, treated weekly with repeated doses of Jo2 (250 *μ*g/kg). Blue: Nuclei staining (Hoechst 33342), green: positive CMH class I signal, white arrow: rat hepatocyte.

Thus, we interpreted this lack of repopulation as a deficit of selective advantage [18]. In literature, several studies used a growth selective advantage by repeating recipient hepatocyte apoptosis with Jo2 while transplanting Jo2 resistant cells: for example, BCl-XL overexpressing mouse hepatocytes (i.e., resistant to jo2) transplanted in a CBA mouse resulted in a 4% mean implantation rate (range from 2 to 6%) [55], and BCl2 over-expressing mouse hepatocytes, more resistant to Jo2 than BCl-XL cells [75], resulted in a mean 30% of repopulation (ranging from 1 to 30%) [76]. We hypothesized that because rat hepatocytes were resistant to Jo2 drug they would get this selective advantage, on a Jo2 treatment repeated model [18]. In fact, repeated administration of Jo2 maintained liver deficiency in Nude mice. We observed [18] that the effects of each weekly Jo2 challenge were equivalent during a 3-week experiment: after each 250 *μ*g/kg body weight dose of Jo2, liver injury could be proven by the increases in serum ALT levels following 24 h. Routine histology showed panlobular necrosis 24 h after the last Jo2 administration, identical to the necrosis seen 24 h after the first Jo2 administration, meaning liver remained sensitive to anti-Fas antibody. Three weeks after transplantation, engraftment rate was determined by immunodetection of the transplanted rat hepatocytes using an anti-rat MHC type I antibody (Figure 3). Engraftment of xenogeneic rat hepatocytes, when transplanted to Nude mice presenting an acute liver failure induced by a single sublethal injection of this anti-Fas antibody, could be improved when mice were further submitted to a weekly repeated Jo2 apoptosis-inducing treatment. In the latter case, engraftment was increased about sevenfold (about 2.4% of repopulation). On genomic analysis comparing Jo2 single dose versus weekly repeated Jo2 treatment in Nude mice receiving rat hepatocytes, the altered pathways suggested a blockade of cell cycle and proliferation (upregulation of cell cycle regulation and downregulation of circadian transcripts), activation of interferon-*γ* pathways, activation of antigen-presenting cells that probably reflects the immune system activation secondary to hepatocyte necrosis and liver injury, and metabolic pathway inhibition confirming liver injury. This overall transcriptome profile might correspond to a selective advantage model where cell cycle blockade occurs in mouse hepatocytes submitted to weekly Jo2 treatment, while natural Jo2 resistance of rat hepatocytes allows them to proliferate [18].

Apart from toxic liver injury, Rhim et al. [19] transplanted rat hepatocytes into a transgenic uPA^+/+^-Nude mice, with high rate (near 100%) of repopulation achieved in 6 to 14 weeks. Presence of rat hepatocytes was confirmed by histology, immunostaining, and rat transferrine measurement.

Weglarz and Sandgren used the same model to determine that hepatocyte entry into DNA synthesis depends on each species and is not influenced by nature of animal recipient and engraftment [74].

For rat hepatocyte transplantation model, Nude mouse seems to be efficient, with complete repopulation in transgenic uPA^+/+^ model, and less important engraftment in other hepatic injury models, comparable to results obtained in similar allogenic models.

### 3.2. Nude Mouse Is a Suitable Human Xenotransplantation Model to Study Engraftment Mechanisms

#### 3.2.1. Human Mature Hepatocyte Transplantation

We recently demonstrated [77] that human hepatocyte transplantation into recipient Nude mice submitted to sublethal and lethal repeated Jo2 liver injury could present a hepatoprotective effect, despite a very poor engraftment rate, lasting up to 7 weeks after transplant. Genomic analysis correlated this lack of engraftment to an absence of selective advantage, cumulated with a paradoxical increased survival rate linked to stimulation of host cell proliferation. These genomic results are in contradiction with those observed with the same treatment protocol and rat hepatocyte transplantation [18]. 

This hepatoprotective effect of mature hepatocyte transplantation was also described by Banas et al. [24]. They generated in vitro human adipose-derived stem cells- (ASC-) derived hepatocytes, and they transplanted these hepatocyte-like cells into female Nude mouse 24 hours after an intraperitoneal injection of 10 *μ*L/20 g of CCl_4_. Woo et al. [23] transplanted hepatocyte-like cells derived from human embryonic stem cell (iPS) to Nude mice and found an hepatoprotective effect, not only on engrafted nodules but also on places far from them, which could be linked to delivered trophic factors (same effect observed after injection of secreted proteins alone). 

Commenting on the very poor engraftment in the repeated Jo2 Nude mouse model [18], it is of common knowledge that even in more immunosuppressed and transgenic models, an important rate of failure of chimerism has been observed. For example, Dandri et al. [28] described a 70%-success rate to raise minimal human hepatocyte engraftment in a uPA^+/+^/RAG2^−/−^ mouse liver, with an all-or-none response for human hepatocyte engraftment. It means that in one-third of their mice, human cells did not engraft at all. They correlated this 30%- failure result with time of prolonged warm ischemia and with poor viability of transplanted cells. These results were confirmed in a model of uPA/SCID mouse where engraftment was successful in a median of 22% (0–87%) of animals, linked with donor age and warm ischemia [78]. Other authors also report failure of chimerism in human-murine models [79].

#### 3.2.2. Human Liver Progenitor Cell Transplantation

Human fetal liver progenitor cells are able to engraft in Nude mouse model, after retrorsine injection followed three weeks later by 30%-partial hepatectomy challenging [22], 50%-partial hepatectomy alone [20], or galactosamine-induced liver deficiency [21]. Engraftment rate range from 0.05–10% [20] and 4-5% [21] to 5–12% [22]. Repopulation yield could be triggered by cotransplantation with fetal liver mesenchymal cells [22] or repeated liver biopsies [20]. 

Interestingly, in these Nude mouse models, human fetal liver progenitor cells not only demonstrate proliferation but also differentiation into both mature hepatocyte and cholangiocyte pathways. Four to six weeks after transplantation, clusters of cells have developed, that have lost their progenitor markers and display morphological and immunohistological markers of mature cells [20–22].

We can compare these results to those obtained by transplanting HepaRG naturally immortalized human liver cell line into SCID/beige mice (SCID-NK cell deficient mice) that were thereafter (first injection began at day one posttransplantation) treated with 0.2 mg/kg Jo2 once a week for 10 weeks [25]. HepaRG cells are bipotent progenitor and in vitro express biologic functions at the same level as primary human mature hepatocytes. They engrafted in Jo2-treated mice liver with a 15–20% repopulation of recipient liver, which is comparable to results obtained with fetal hepatoblast transplanted into Nude mice [22]. 

 The results altogether suggest that it is of most importance to pay attention to chronology (to avoid transplantation preceding liver injury), to be the closest to human clinical scene.

## 4. Nude Mouse Is Not a Suitable Xenotransplantation Model for the Creation of Humanized Liver

Most of maximal human cell engraftment was described on two transgenic models with highly immunosuppressed mouse models, uPA^+/+^ mice and Fah^−/−^ mice. This engraftment could become sufficient to talk of “humanized liver,” with human hepatic functions and human pharmacological proprieties [12–14, 80]. 

The first is the uPA^+/+^ mouse model. To our knowledge, there was no publication of human liver cell transplanted in a Nude mouse uPA model. 

The second transgenic Fah^−/−^ mouse model was used by Azuma et al. [27] in Fah^−/−^ Nude mice model to create humanized liver, but they did not obtain sufficient human cell engraftment in this model and had to use a more immunosuppressed transgenic model (Fah^−/−^/Rag2^−/−^-/Il2rg^−/−^- mice) reinforced by uPA-adenovirus administration intravenously 24–48 hours before human hepatocyte transplantation, associated to NTBC withdrawal over five days after transplantation.

To our knowledge, no success in humanized livers in Nude mice has been reported, most likely because of an insufficient immunosuppression of this mouse.

In other uPA^+/+^ immunosupressed mouse models, such as Rag2^−/−^ mice, human hepatocytes can engraft, although at lower rate than murine hepatocytes [79, 81]. In such a model, Dandri et al. [28] reported 8 weeks after human hepatocyte transplantation a 2 to 10% of repopulation by human cells. In the Fah^−/−^ mouse model, human hepatocytes could repopulate Fah^−/−^/Rag2^−/−^/Il2rg^−/−^ mouse liver with a range from 5 to 34%, 12 weeks after transplant [26].

But these two models have specific disadvantages, directly linked with their concept; uPA^+/+^ mice, because of their inborn metabolic abnormally, have a poor breeding efficiency, a quite narrow window in time to perform transplantation (on neonatal age, between1 to 3 weeks of age, before they die of severe bleeding), and a renal disease that still exists despite hepatocyte transplantation [19, 27, 59, 60]. They can return to wild type by inactivation of the gene [59]. Moreover, there is a continuous and progressive hepatic parenchyma injury, possibly via activation of plasminogen and modified activity of matrix metalloproteinase: thus, this modified metabolism can interfere with liver cell growth and distort a physiopathologic model [30].

Fah deficient mice also have inconveniences: their metabolic pathway leads to development of liver hepatocellularcarcinomas [82], requiring treatment by NTBC-diet repeated cycles to prevent tumor formation and maintain long-term survival. This diet cancels the natural selective advantage that triggers xenogenic cells proliferation and can give possible bias in results interpretation. To increase human hepatocyte repopulation efficiency, some teams use a transfection of uPA gene, which adds the same issues as encountered in uPA^+/+^ model [27].

Another strategy is represented by liver suicide model: it consists to transfect recipient liver hepatocytes with an apoptotic gene; this gene being under the control of herpes virus type 1 thymidine kinase (HSV TK): it can be activated by administration of Gancyclovir, and thus could destroy specifically cells targeted with this suicide gene [83]. 

Hasegawa et al. [30] obtained an NOG (NOD/SCID/Il2Rg^−/−^)  mouse expressing HSV TK transgene in their liver. By inducing apoptosis of liver recipient cells five days before transplantation of human hepatocyte, they observed a high index of repopulation (average up to 43%), correlated with elevated human albumin in plasma, and functional human hepatocyte. 

Douglas et al. [31] who used uPA-SCID model optimized it to achieve total replacement of uPA^+/+^ hepatocytes by human hepatocytes, by associating mouse liver suicide to uPA-SCID model, so that Ganciclovir treatment could induce conditional selective murine hepatocyte death in humanized SCID-uPA mouse liver. But unexpected, mice survival was not increased by humanized liver, whatever the repopulation index (32 to 87%). 

As illustrated by the results of Douglas et al, the limits of the humanized liver reside intrinsically in the principle of xenogenic transplantation: whatever the models, even in the best repopulation performance such as uPA^+/+^ or Fah^−/−^ transgenic mouse, xenogenic murine models gave best engraftment results compared to human cells transplantation [29]. Interestingly, it does not seem possible to avoid a percentage of nearly complete failure of human cells engraftment in mouse liver, for each experiment, even in the studies reporting very high repopulation index [28, 79]. 

This disparity between animals of the same study could be due to imperfect immunosuppression [84] or inadequation between murine and human metabolism [74, 81]. 

In favor of the first hypothesis, Tateno et al. [85] showed that when human hepatocyte engraftment in uPA-SCID mouse results in more than 50% repopulation, this high repopulation index leads to death of recipient. This mortality can be corrected by a treatment that blocks human complement factor activity.

In favor of the second hypothesis are the results of Su et al. [26] of a failure to induce chimerism in a Fah^−/−^/Rag2^−/−^/Il2rg^−/−^ mouse transplanted with human hepatocyte, in 6 over 14 mice despite an immunosuppressive drug (FK506). Also in favour of this hypothesis is the observation that mouse survival was not increased by humanized liver, whatever the repopulation index (32 to 87%), in the uPA-SCID model of liver failure challenged with liver suicide gene activation [31]. There seems to be an incompatibility directly linked to animal species differences: although human and murine cells can create narrow cellular junctions, morphologically and architecturally subnormal links confirming integration of xenogenic cells but human cells will develop unexplained glycogen storage or steatosis anomalies [81, 86]. 

Therefore, the hypothesis that humanized liver would allow performing experiments not feasible on humans thus helping to predict pharmacotoxicological and pathobiological effects in humans needs additional demonstration. But, even in this case, transgenic models would mimic only limited aspects of human clinical liver failure, and one should be aware that humanized liver may not be transposable to real human physiology. 

As a last comment, the use of an immunodeficient animal model could also be a bias in these liver cell transplantation studies. In fact, immune cells could modulate liver regeneration [87–89]. Their absence in immunodeficient animals could modify the liver response to acute injury (demonstrated by Strick-Marchand et al. [90]) and to chronic injury, as well as engraftment of hepatic cells. In the other hand, the use of immunosuppressive treatment for transplanted nonimmunodeficient animal models could also interact with liver physiology [91, 92] and become another bias. 

This raises the question of the bias of using xenogenic models, with respect to the risk taken by xenogenic animal models not reflecting human physiology. 

## 5. Conclusion

As a conclusion, Nude mice can sustain various liver injuries and are good recipients for xenogenic rodent hepatocyte transplantation. They remain an acceptable model for human hepatocyte engraftment and for human hepatic progenitor cell transplantation, by exploring the beneficial environment allowing the differentiation of the latter into mature hepatocyte as well as biliary cells. The low immunosuppressive background of nude mice is both an advantage, with easy breeding conditions, and disadvantage, with difficulties to raise humanized liver. They are certainly an interesting model to study liver regeneration mechanisms in a context of human clinical liver deficiency situations. 

## Figures and Tables

**Table 1:** Literature review of xenogenic liver cell transplantation in immunodeficient mouse models.

Reference	Year	Recipient animal	Induced liver deficiency model	Transplanted cells	Duration of the study	Engraftment efficiency (%) and outcome	Precised mouse type
Vidal et al. [18]	2008	Nude mice	Jo2 250 *μ*g/kg weekly	Sprague-Dawley *rat* hepatocyte	3 weeks	4.2%	Balb/c Nude mice
Rhim et al. [19]	1995	uPA-Nude mice	uPA	Sprague-Dawley *rat* hepatocyte	8 weeks	Reach 100%	uPA mice crossed with Swiss athymic nude (nu/nu) mice
Mahieu-Caputo et al. [20]	2004	Nude mice	PHx 50%	Human fetal hepatoblast (11–13 WG)	6 weeks	0.05 to 5% (increased if liver biopsies were repeated)	Athymic mice
Nowak et al. [21]	2005	Nude mice	GalN IP 0.7 g/kg 36 h prior to transplantation	CD117+/CD34+/Lin-human fetal liver cell (6–10 WG)	4 weeks	4-5% (differentiation into mature hepatocytes and cholangiocytes)	C57 Black/nude mice
Joshi et al. [22]	2012	Nude mice	Retrorsine 70 mg/kg and PHx 30%	Human fetal hepatocyte/liver mesenchymal cell	4 weeks	12% (MSC enhanced liver repopulation)	C57BL/6 nude mice
Woo et al. [23]	2012	Nude mice	CCl_4_ 10 *μ*L/20 g	Hepatocyte-like cells derived from human embryonic stem cell (iPS)	5 weeks	20–23% (hepatoprotective effect obtained by delivering trophic factors)	Balb/c nude mice
Banas et al. [24]	2009	Nude mice	CCl_4_ 10 *μ*L/20 g	Adipose-derived stem cells derived hepatocyte-like cells	One day	? (Protect against liver damage)	Balb/c nude mice
Jiang et al. [25]	2010	SCID beige mice	Jo2 0.2 mg/kg weekly	Human HepaRG cell line	10 weeks	15–20%	SCID beige mice
Su et al. [26]	2011	Fah^−/−^Nod/SCID mice and FK506 treatment	Fah	Human hepatocyte	6 weeks	0.6–18% (chimerism successful in 8/14 mice)	Fah^−/−^Nod/SCID mice
Azuma et al. [27]	2007	Fah^−/−^/Rag2^−/−^/Il2Rg^−/−^ mice	uPA adenovirus transfert	Human hepatocyte	8 to 12 weeks	>1% for 17/73 mice >30% for 7/17	Fah/Rag2/Il2rg triple mutants
Dandri et al. [28]	2001	uPA/Rag2^−/−^ mice	uPA	Human hepatocyte	4 weeks	2–10% (But no engraftment in 3/10 mice)	uPA/Rag2^−/−^ mice
Haridass et al. [29]	2009	uPA^+/−^/rag2^−/−^/*γ*c^−/−^	uPA	Mouse and human hepatocyte and fetal liver cell	3 months	Mouse hepatocyte 46% Mouse fetal cells 5–12% Human hepatocyte 10% Human fetal cells 2.7%	uPA^+/−^/rag2^−/−^/*γ*c^−/−^ mice
Hasegawa et al. [30]	2011	TK-NOG mice	Ganciclovir	Human hepatocyte	Up to 8 months	43%	Nonobese diabetic/SCID/IL2R*γ* ^−/−^ mice
Douglas et al. [31]	2010	vTK-SCID/uPA	Hepatic failure induced by Gancyclovir (for hepatocyte bearing HSVtk plasmide)	Human hepatocyte	8 weeks	No survival of mice	SCID/uPA mice

WG: weeks of gestation, PHx: partial hepatectomy, GalN: Galactosamine, uPA: uroplasminogen activator, Fah: fumarylacetoacetate hydrolase, MSC: mesenchymal stem cells, and CCl_4_: carbon tetrachloride.
